# Associations between PACS symptoms and risk factors among Long Haulers in the Rutgers Post-COVID Recovery Program

**DOI:** 10.1093/geroni/igab046.3670

**Published:** 2021-12-17

**Authors:** Alice Dawson, William Hu

**Affiliations:** 1 Rutgers University--New Brunswick, New Brunswick, New Jersey, United States; 2 Rutgers University, New Brunswick, New Jersey, United States

## Abstract

At least 2/3 of people with mild to moderate COVID-19 infection will experience long-haul COVID symptoms that persist for weeks or months, however, risk factors that modify the likelihood that one develops these symptoms are unknown. Patients referred to the Post-COVID Recovery Program at Rutgers in New Brunswick (n= 108) through primary care referral or self-submitted online request and experiencing a wide variety of Post-Acute COVID-19 Syndrome (PACS) symptoms were stratified by those without self-reported cognitive complaints (n=54), those with self-reported cognitive complaints who scored well on cognitive testing (n=29), and those with self-reported cognitive complaints who scored poorly on cognitive testing (n=25). Comparisons between groups were made using ANOVAs and Chi Squared: for COVID-19 disease severity, COVID-19 disease treatment, comorbid COVID-19 symptoms during infection, comorbid PACS symptoms post-infection, pre-existing health conditions, levels of depression and anxiety, level of fatigue, and social determinants of health (access to healthcare, economic stability, housing stability). Preliminary analyses indicated that whereas people without complaints were normally distributed according to age (p>0.200 for Kolmogorov–Smirnov test), people with complaints and deficits were skewed towards the older age group (p<0.001 for K-S test) suggesting age to be a risk factor for cognitive impairment in PACS. Participants that reported cognitive complaints also reported increased symptoms of depression, anxiety, and fatigue, compared to participants without cognitive complaints. These data provide insight into associations between PACS symptoms and risk factors relevant in understanding this novel disease.

